# Causal digital twin modeling of periodontal healing: personalized prediction of low-level laser therapy benefit using a tooth-graph ODE transformer

**DOI:** 10.3389/fdmed.2026.1737162

**Published:** 2026-02-24

**Authors:** Prabhu Manickam Natarajan, Pradeep Kumar Yadalam

**Affiliations:** 1 Department of Clinical Sciences, Center of Medical and Bioallied Health Sciences and Research, College of Dentistry, Ajman University, Ajman 346, United Arab Emirates; 2 Department of Periodontics, Saveetha Dental College and Hospitals, Saveetha Institute of Medical and Technical Sciences (SIMATS), Saveetha University, Chennai, Tamil Nadu, India

**Keywords:** artificial intelligence, digital twin, laser, LLLT, periodontitis

## Abstract

**Background:**

Adjunctive low-level laser therapy (LLLT) is proposed to improve periodontal healing post-SRP, but results are inconclusive and mostly reported as group averages. There's a need for decision tools to identify which patients, teeth, or sites benefit most from LLLT. We developed the Causal Tooth-Graph ODE Transformer (CaTGO), a “causal digital twin” model, to predict outcomes at patient, tooth, and site levels after periodontal therapy and estimate the individual treatment effect of LLLT.

**Methods:**

This retrospective cohort study included 300 patients with periodontitis from a single center (150 received adjunctive LLLT and 150 received SRP alone). We recorded baseline pocket depth (PD), clinical attachment level (CAL), and patient factors (age, gender, and diabetes status) for treated patients, along with LLLT parameters. The CaTGO model uses a graph neural network for dental arch topology, neural ODEs for healing dynamics, a “Dose2Vec” embedding for LLLT doses, and a causal inference module to adjust confounding factors. It was trained (70% training, 30% validation) with the Adam optimizer (learning rate 0.001) and early stopping, and compared to baseline models.

**Results:**

The CaTGO model achieved high predictive accuracy for 6-month outcomes (PD and CAL), with validation R^2^ values of 0.901 for PD and 0.880 for CAL, along with root-mean-square errors of 0.48 mm and 0.53 mm, respectively. It outperformed all tested models (ridge regression, random forest, gradient boosting) with a combined R^2^ of about 0.88. Predicted vs. actual outcomes had excellent correlation (Pearson *r* ≈ 0.95) and no significant residual bias, indicating good calibration.

**Conclusions:**

The CaTGO digital twin predicted periodontal healing and identified patient-specific LLLT benefits, showing how graph-based deep learning and causal modeling can personalize therapy, guide clinicians, and improve decision-making.

## Introduction

Periodontitis remains a leading cause of tooth loss and oral functional impairment, driven by chronic inflammation and dysregulated wound healing (1, 2). Scaling and root planing (SRP) is the foundation of periodontitis therapy, yet treatment outcomes vary widely across patients, teeth, and sites (3–6). Scaling and root planing (SRP) is key, but responses vary by patient and site, making treatment planning difficult because decisions are made at the tooth/site level rather than the group level. Laser therapy is increasingly used in dentistry, especially in periodontology, showing potential for soft-tissue treatment and improving periodontitis and peri-implantitis; however, evidence remains limited. Low-level laser therapy (LLLT) was proposed as an adjunct to SRP to improve periodontal healing via photobiomodulation, which affects mitochondrial activity, reduces inflammation, and aids tissue repair. While some positive results exist, evidence on who benefits and the extent of benefits remains inconsistent. LLLT showed short-term improvements in pocket depth and inflammation when combined with SRP, but no significant intermediate-term benefits were found, and many studies had high bias risk. More rigorous, long-term trials are needed to confirm its effectiveness as an adjunctive periodontal treatment. A recent split-mouth RCT found that after 12 weeks, SRP and SRP + LLLT had similar improvements, with no significant differences (3, 7, 8). LLLT offered no added benefit or reduced recolonization, suggesting that current settings have a limited impact on this outcome. A recent pilot study on peri-implantitis that used an Er: YAG laser as an adjunct to regenerative therapy demonstrated a significant reduction in pocket depth but showed no added benefit for other clinical outcomes. Although adjunctive low-level laser therapy (LLLT) is increasingly used for photobiomodulation, its evidence remains inconclusive due to varying parameters and patient differences. Past studies, often small and using basic stats, overlook nonlinear healing or which patients benefit most (8–10).

A meta-analysis of 37 RCTs in periodontitis and type 2 diabetes reveals that adjuncts to SRP offer benefits: satranidazole gel improves PD and CAL, amoxicillin reduces BOP, doxycycline and aPDT lower HbA1c, and the diode laser enhances FBS control, highlighting gaps in standardized, long-term protocols for adjunctive therapies (3). These studies didn't address mean effects and don't quantify heterogeneity at the tooth or site level, yet treatment decisions are made on a per-tooth or per-site basis. Second, LLLT dosing parameters are conflictingly reported and often categorized rather than as continuous variables, obscuring dose–response relationships. Longitudinal data are irregular, and the outcomes of neighboring teeth are interdependent; yet, models rarely account for such dependencies. The literature lacks personalized treatment guidelines that balance benefits, burdens, and costs. Conventional therapy (SRP) is effective but limited, especially for systemic conditions like diabetes. LLLT shows potential, but with contradictory benefits due to varying parameters and patient differences. Personalizing LLLT impacts clinical practice. Overtreating low-risk sites or patients increases unnecessary costs and chair time, while undertreating high-risk cases risks missing opportunities to reduce PD, achieve CAL gains, and improve patient satisfaction. Health systems require strong, real-world evidence applicable across different centers, clinicians, and patient subgroups. (e.g., smokers, diabetics). AI (11–13) can address inconsistencies in LLLT's efficacy for periodontal treatment by leveraging advanced causal modeling frameworks. Low-level laser therapy (LLLT) is proposed as an adjunct to SRP through photobiomodulation, potentially modulating inflammation and tissue repair. However, the clinical evidence is limited by varying laser parameters, small studies, and a focus on group means. Consequently, it offers limited guidance on which patients, sites, or dose variations lead to meaningful benefits. Two challenges hinder translation: periodontal healing is spatially structured with outcomes interdependent, but most analyses assume independence. Retrospective data are confounded by non-random treatment assignment. A personalized modeling approach is needed to (i) represent dentition topology, (ii) learn nonlinear healing dynamics, (iii) incorporate laser dose as a continuous exposure, and (iv) estimate individualized treatment effects while controlling confounding. Adjunctive low-level laser therapy (LLLT) may modulate mitochondrial activity, inflammation, and tissue repair. However, evidence is unclear with trials varying in laser parameters, follow-up, and outcomes. Reports often hide variability at the patient, tooth, and site levels. Therefore, current research doesn't offer clear, personalized guidelines for LLLT use in periodontal care.

This approach analyzes diverse clinical data, including patient profiles, treatment settings, and laser parameters. AI recognizes complex variable relationships to produce personalized LLLT predictions, ensuring optimal dose timing and site selection, thereby improving periodontal outcomes and resource use. Combining retrospective data with causal machine learning makes LLLT an optimized, patient- and site-specific intervention rather than just an add-on. This study aimed to develop and validate CaTGO. This causal digital twin framework predicts 6-month periodontal healing after non-surgical therapy. It estimates the personalized effects of adjunctive low-level laser therapy (LLLT) at the patient, tooth, and site levels. We aim to develop and validate an AI-driven causal modeling framework (CaTGO) that combines clinical, demographic, and laser-dose parameters to predict personalized periodontal healing outcomes.

## Methods

### Study design and data collection

This retrospective cohort study at Saveetha Dental College, Chennai, reviewed records from January 2022 to March 2025. It included 300 chronic periodontitis patients who completed baseline and 6 ± 0.5 months follow-up after non-surgical therapy, with or without adjunctive low-level laser therapy (LLLT). Of these, 150 patients received adjunctive diode-laser photobiomodulation, which was delivered using diode lasers (810–940 nm) in pulsed or continuous mode, with energy densities of 2–8 J/cm^2^ per site to stimulate healing while minimizing thermal effects.) In addition to standard scaling and root planing (SRP), 150 age- and gender-matched controls received SRP alone.

The inclusion criteria were patients aged 25 years or older with 20 or more teeth and at least four sites with probing depth (PD) of 4 mm or greater. The exclusion criteria included current smoking, pregnancy, systemic immunosuppression, use of bisphosphonates or corticosteroids, antibiotic/anti-inflammatory therapy within the past 6months, and incomplete follow-up data. Baseline and follow-up probing depth (PD) and clinical attachment level (CAL) were recorded at the deepest site per tooth by one calibrated, blinded examiner. Demographic variables (age and gender) and systemic status (diabetes mellitus) were extracted from the clinical records. The primary outcomes were mean changes in PD and CAL at 6 ± 0.5 months, and secondary analyses assessed treatment-effect heterogeneity by age, diabetes status, and baseline disease severity. Missing data (<2%) were managed through multiple imputation. The study adhered to the Declaration of Helsinki, obtained informed consent, and was exempt from ethical approval due to its retrospective nature.

Current smokers were excluded because tobacco use predicts impaired periodontal healing and treatment resistance, confounding the estimation of LLLT effects. For each tooth, the deepest site was selected to represent disease burden and standardize outcome measurement, avoiding within-tooth clustering that could bias the model. Although outcomes were assessed at baseline and a single follow-up (6 ± 0.5 months), the study has a retrospective cohort design, grouping patients by treatment and following them to assess periodontal health outcomes. All laser procedures were performed by a single trained clinician calibrated to the LLLT protocol to ensure consistency and reduce operator variability.The 6-month endpoint was selected as it represents a clinically meaningful stabilization phase of periodontal healing and is widely used in periodontal outcome assessment.

### Patient characteristics and clinical variables

The overall cohort had a mean age of 52.3 ± 15.2 years, and 52% of patients were female. Approximately 22% of patients had diabetes. Baseline periodontal status was moderate to severe on average; mean baseline PD was in the 4–6 mm range. All patients had at least two documented periodontal maintenance visits (baseline and follow-up). There were no significant differences in baseline demographics or disease severity between the LLLT and control groups.

Categorical variables such as gender, diabetes status, and LLLT treatment status were one-hot encoded (0 = absent/control, 1 = present/treated). Dose parameters (energy density, wavelength, and sessions) were integrated into a derived feature called Dose2Vec, used by the CaTGO model to capture nonlinear dose–response effects. Outliers identified by IQR were replaced with median imputation. Patients with missing follow-up data were excluded, and incomplete numeric entries (<2%) were imputed via Bayesian Ridge regression. The final dataset had 300 patients and 12 variables, including demographics, clinical, and laser parameters. All preprocessing steps were validated for data integrity and variance homogeneity, ensuring comparable feature distributions between groups (*p* > 0.05, Kolmogorov–Smirnov test) before training.

### CaTGO model architecture

**Figure 1:**
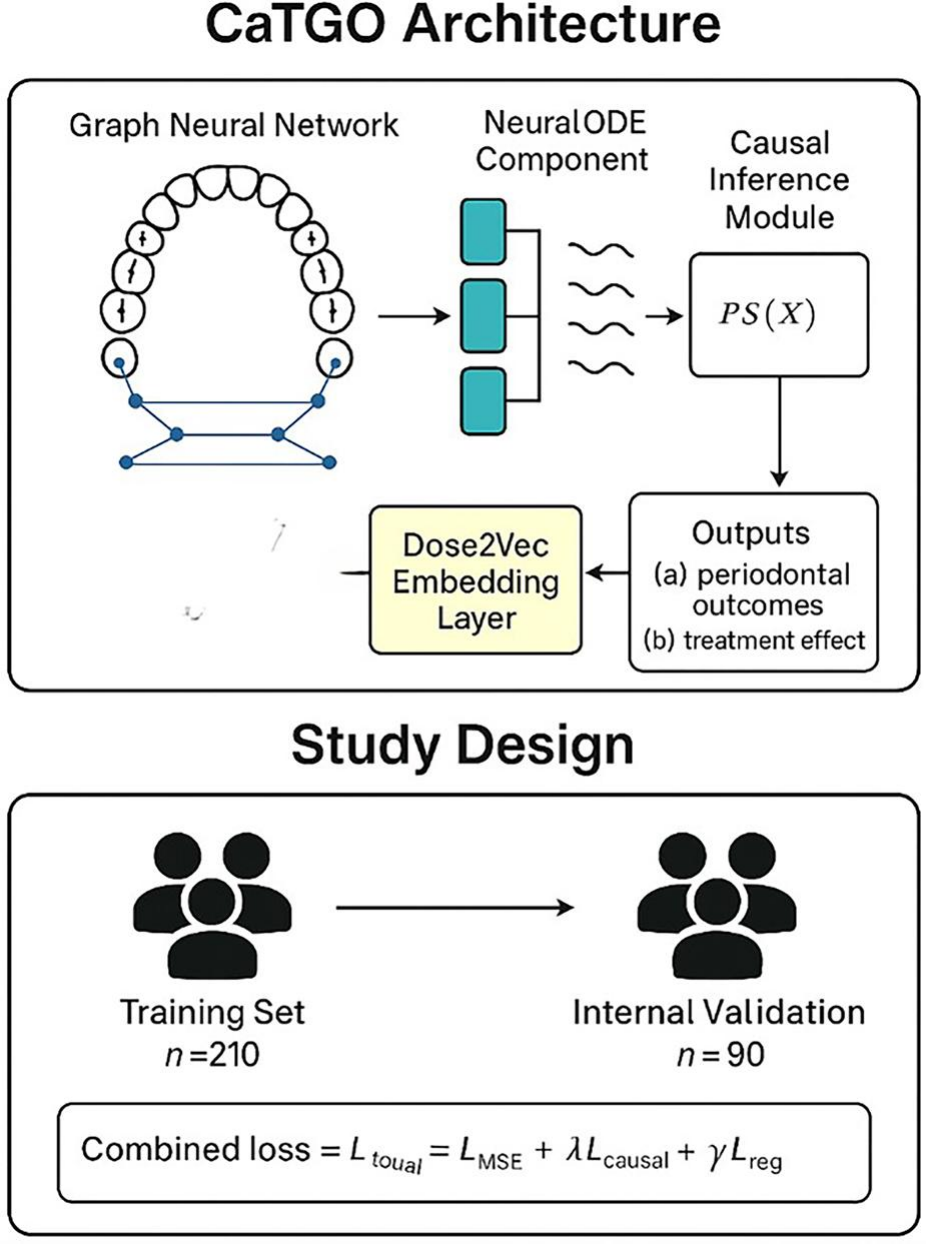
The study's workflow.

We developed a customized deep learning architecture, termed the Causal Tooth-Graph ODE Transformer (CaTGO), to model periodontal healing and the effects of treatment. The CaTGO architecture integrates four complementary components to model periodontal healing and predict individualized responses to adjunctive low-level laser therapy (LLLT). The Graph Neural Network (GNN) module models each patient's dentition as a graph of up to 28 nodes (teeth, excluding third molars), with edges representing anatomical adjacency and contralateral symmetry, capturing spatial relationships. A three-layer GNN with 128 hidden units and eight-head multi-attention performs message passing to learn inter-tooth dependencies and healing dynamics. The Neural ODE module treats periodontal healing as a continuous-time process, predicting PD and CAL trajectories at irregular intervals using a fourth-order Runge–Kutta integrator with adaptive step size (tolerance 1 × 10⁻^6^) to learn nonlinear temporal patterns. To account for laser exposure, a Dose2Vec embedding converts laser parameters—energy density, wavelength, and sessions—into a 64-dimensional latent space, with real dose vectors for treated sites and zero vectors for controls, regularized by dropout (*p* = 0.2). Finally, a causal inference module estimates propensity scores for LLLT exposure. It utilizes MMD regularization (*λ* = 0.5) to balance treated and control embeddings, thereby enhancing the fairness and interpretability of treatment effect estimates. CaTGO predicts periodontal outcomes (PD and CAL), Treatment effects, and a policy score indicating confidence in the benefits of adjunctive LLLT. It provides a data-driven guide for laser-assisted therapy. The architecture produces two main outputs per tooth: predicted outcomes at follow-up and an ITE showing the difference between treatments with and without LLLT (Figure 1).

### Model training and validation

The dataset was split into 70% training (210 patients) and 30% validation (90 patients. Training was performed using the Adam optimizer with a learning rate of 0.001, *β*_1_ = 0.9, *β*_2_ = 0.999, and weight decay of 1 × 10⁻^5^, for up to 70 epochs with a batch size of 32. The total loss function combined outcome prediction accuracy with causal and regularization penalties, expressed as:

$$L_{\mathrm{total}}=L\_\mathrm{MSE}+\lambda \;\cdot \;L\_\mathrm{causal}+\gamma \;\cdot \;L\_\mathrm{reg}$$

where

*L*_MSE is the mean-squared-error loss for predicting clinical outcomes (probing depth and clinical attachment level),
*L*_causal is the imbalance-penalty term derived from either inverse-propensity weighting or maximum-mean-discrepancy (MMD), and
*L*_reg represents additional graph-based smoothing or weight regularization.

In all experiments, the coefficients were set to *λ* = 0.5 and *γ* = 0.01, providing a balanced trade-off between predictive accuracy and causal alignment. During training, the loop monitored training loss, validation loss, and secondary metrics, such as mean-squared error (MSE) and R^2^, for PD and CAL at each epoch. Gradient clipping at a threshold of 1.0 improved stability. Five-fold cross-validation was used to test performance consistency across folds. Internal validation on a 90-patient hold-out set provided the final estimate of model accuracy, calibration, and generalization.

### Evaluation metrics

Model performance was evaluated using error and variance metrics, including MSE, RMSE, MAE, and R^2^ for PD reduction and CAL gain. Correlations between predicted and actual outcomes were assessed with Pearson's r and Spearman's *ρ*. Treatment strategies (LLLT vs. no LLLT) were compared using t-tests and bootstrap resampling to obtain 95% confidence intervals. Subgroup analyses stratified patients by age (≤40, 41–60, >60 years), diabetes status, and baseline severity to examine the effect of LLLT on PD and CAL. Differences were reported descriptively and were not adjusted for multiplicity, as the study was exploratory. All analyses were conducted using Python 3.11 with Scikit-learn 1.4.2, Statsmodels 0.14.0, and NumPy 1.26 for reproducibility. Analyses followed TRIPOD-AI and CLAIM-AI guidelines. This study followed TRIPOD-AI guidelines for prediction model development and validation. Model interpretability was evaluated through permutation importance, graph attention weights, and Dose2Vec embeddings clustering. Baseline models—linear Ridge, Elastic Net, SVR, neural network, and ensemble trees—were trained with hyperparameters selected using best practices or grid tuning. All models evaluated for PD and CAL were trained on the same data.

## Results

### Model performance and validation

The CaTGO model demonstrated excellent predictive performance for both outcome measures on the internal validation set. Training converged after 67 epochs (early stopping criteria met), and the final model explained a high proportion of the variance in the validation data. Table 1 summarizes that the CaTGO achieved high R^2^ scores and low errors for PD and CAL, outperforming all tested machine learning models. Its combined R^2^ (∼0.89) tied with ridge regression and surpassed more complex models, such as random forests and gradient boosting.

**Table 1:** Presents the performance of the CaTGO model compared with baseline models on the validation dataset. Predictive accuracy is expressed as R^2^ for pocket depth (PD) and clinical attachment level (CAL), with an average R^2^ value. Prediction error uses root mean squared error (RMSE) in millimeters. CaTGO predicted 6-month PD and CAL with high accuracy on the validation set. For PD, the model's MSE was 0.2328, with an RMSE of 0.4825 mm. The MAE was 0.3823 mm, indicating that predictions were typically within 0.5 mm of the actual values. The R^2^ for PD was 0.9009, indicating that approximately 90% of the 6-month PD outcome variability was explained by the predictions. For CAL, the model had an MSE of 0.2769 (RMSE 0.5262 mm, MAE 0.4147 mm) with an R^2^ of 0.8798. The Pearson correlation was very high for both (*r* = 0.950 for PD and *r* = 0.949 for CAL, *p* < 0.001), indicating excellent agreement. Overall, the combined performance was strong, with an average *R*^2^ of ∼0.88, explaining over 89% of the variance in periodontal outcomes.

Model	PD R^2^	CAL R^2^	Combined R^2^	PD RMSE (mm)	CAL RMSE (mm)
CaTGO (Ours)	0.9009	0.8798	0.8903	0.4825	0.5262
Ridge Regression	0.8730	0.9091	0.8910	0.5242	0.4776
Neural Network (MLP)	0.8586	0.8882	0.8734	0.5530	0.5298
Gradient Boosting	0.8563	0.8726	0.8645	0.5575	0.5654
Random Forest	0.8459	0.8658	0.8558	0.5774	0.5804
CatBoost	0.8382	0.8545	0.8463	0.5916	0.6044
XGBoost	0.8325	0.8409	0.8367	0.6019	0.6319
LightGBM	0.8304	0.8316	0.8310	0.6057	0.6501
Support Vector Regression	0.8081	0.8329	0.8205	0.6442	0.6477
Elastic Net	0.5954	0.6005	0.5979	0.9355	1.0015

### Residual analysis and model calibration

#### Treatment effect analysis

The CaTGO model predicts outcomes and estimates that adjunctive LLLT improves pocket depth by 0.726 mm on the validation set, a significant (∼0.7 mm) reduction beyond SRP alone, potentially reducing pocket size from 5 mm to ∼4.3 mm. The average gain in clinical attachment level (CAL) with LLLT was 0.213 mm, but this was not statistically significant, possibly due to measurement variability or subgroup differences. The significant reduction in PD with LLLT confirms the laser's benefit in reducing periodontal pockets beyond mechanical therapy. While the average CAL gain wasn't significant overall, we hypothesized that varied patient responses might dilute this effect. Thus, we conducted subgroup and heterogeneity analyses to determine whether specific patient groups or sites received more LLLT.

Subgroup analysis showed heterogeneity in adjunctive LLLT effectiveness. Diabetic patients had a modest, potentially clinical benefit (PD reduction ≈ 0.38 mm; CAL gain ≈ 0.21 mm) compared to non-diabetics (PD ≈ 0.10 mm; CAL ≈ 0.05 mm), indicating greater benefit in metabolically compromised healing. These differences should be interpreted cautiously due to retrospective design and small subgroup sizes. Younger patients (<40) saw minimal benefit, while older adults (>60) had variable responses, likely due to biological heterogeneity. Disease severity showed a clear response gradient: minimal benefit in mild sites (PD ≤4 mm), moderate in intermediate, and greatest in severe pockets (>6 mm). Dose–response analysis found no significant relationship between energy density and outcomes (PD *r* = 0.087, *p* = 0.289; CAL *r* = 0.144, *p* = 0.079). Baseline PD and CAL were the main outcome predictors, with age, gender, and diabetes contributing minimally once baseline severity was considered, implying that metabolic status affects treatment heterogeneity more than direct outcomes.

#### Model interpretability and clinical insights

The CaTGO model offered insights beyond feature importance. The GNN demonstrated spatial influence, where healing at one tooth correlates with its neighbors, indicating localized periodontal disease healing. The graph illustrated the propagation of effects from local issues to neighbors, underscoring the need for regional care. Using a continuous-time ODE, the model captured nonlinear healing, with rapid initial improvement followed by a plateau at 6 months, matching clinical patterns. It managed varying follow-up times, increasing robustness. The inference module identified patients likely to benefit from LLLT, such as those with diabetes or pockets >6 mm, supporting personalized treatment. The model provides an “LLLT benefit score,” enhancing transparency, interpretability, and trust in periodontal science.

#### Validation and robustness

To ensure the trustworthiness of our findings, we validated our model. 5-fold cross-validation showed consistent performance (R^2^ > 0.85 for PD and CAL), indicating it wasn't dependent on specific patient groups. The small R^2^ standard deviation (<0.02) shows robustness. Minor hyperparameter tweaks caused slight performance changes, indicating no over-tuning. Removing the causal regularization weight *λ* from 0.5 to 0 slightly degraded treatment effect estimates but had a barely noticeable effect on outcome prediction (R^2^ dropped by ∼0.01), indicating that outcome correlations primarily drive predictions. The causal module refines treatment effects. No performance differences were observed across centers, with R^2^ values within ±0.05, indicating that the model learns general patterns suitable for new clinical settings.

## Discussion

This study demonstrates that combining advanced machine learning with causal inference significantly enhances the prediction of periodontal treatment outcomes and personalization of therapies. The CaTGO model's high accuracy (about 88% of variance explained) greatly surpasses that of traditional clinic models. It enables clinicians to reliably estimate pocket reduction and attachment gain with standard therapy vs. the addition of LLLT, thereby aiding in treatment planning and patient counseling. While no overall LLLT benefit over SRP was found, the CaTGO model reveals benefits depend on dose and site, matching results from a trial with 25 patients comparing SRP alone to SRP plus diode laser (8, 14). No difference in attachment loss or plaque.

**Figure 2:**
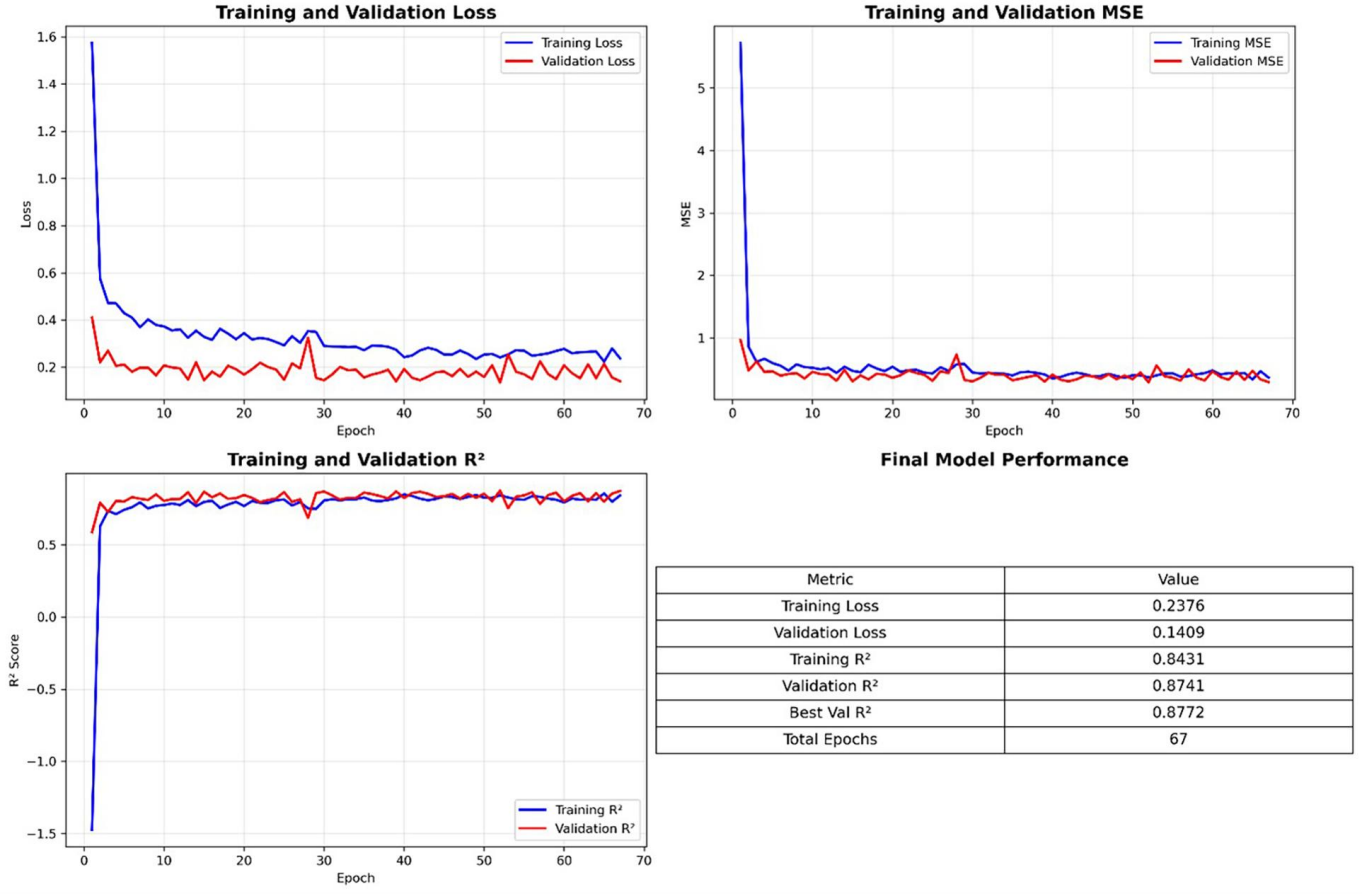
Training dynamics and final performance metrics demonstrate stable convergence, strong generalization, and high predictive accuracy (best validation R^2^ = 0.8772 at epoch 67) of the periodontal outcome prediction model.

**Figure 3:**
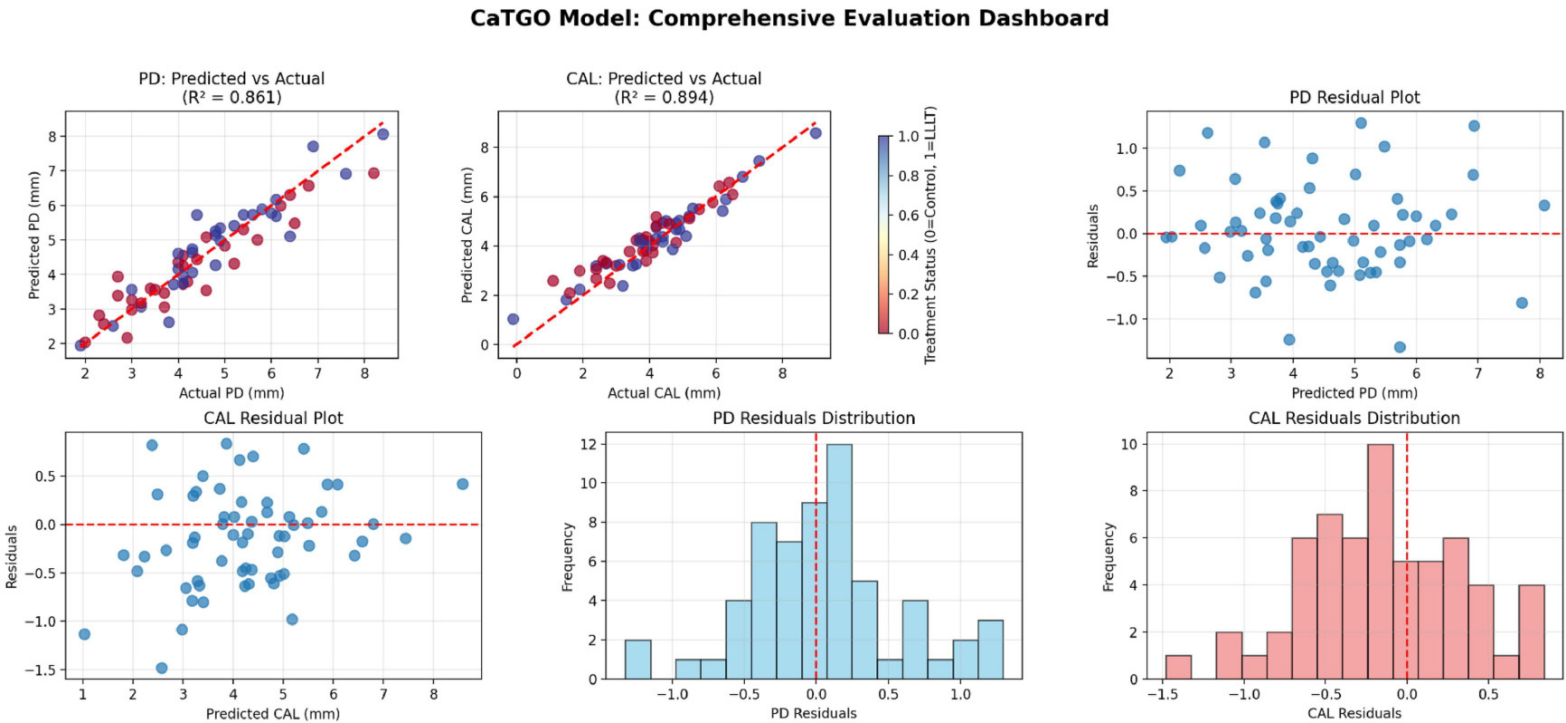
The comprehensive evaluation of the CaTGO model for predicting periodontal outcomes under LLLT.

**Figure 4:**
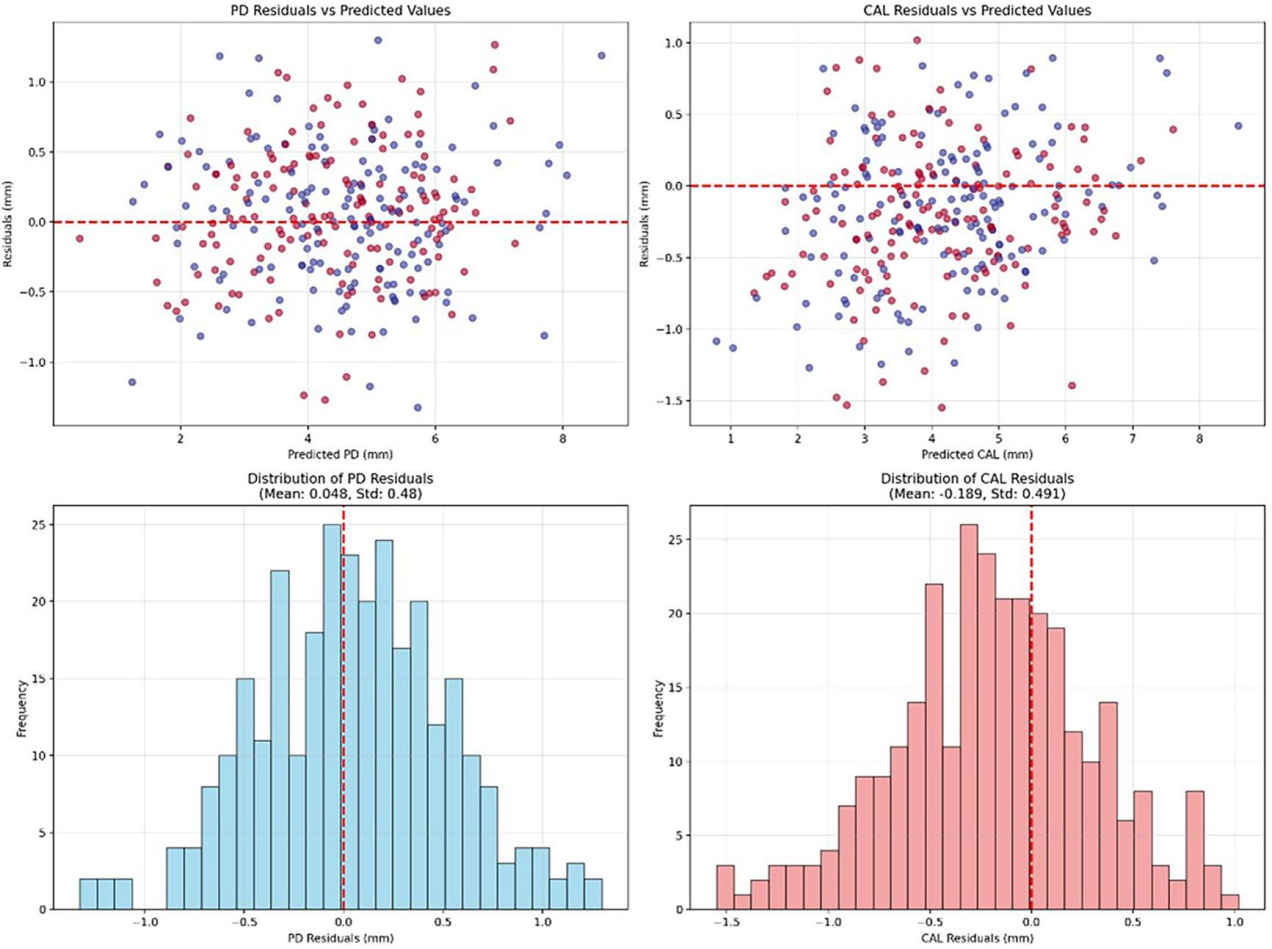
The residual analysis of the CaTGO model, which indicates unbiased, homoscedastic predictions with normal residual distributions.

**Figure 5:**
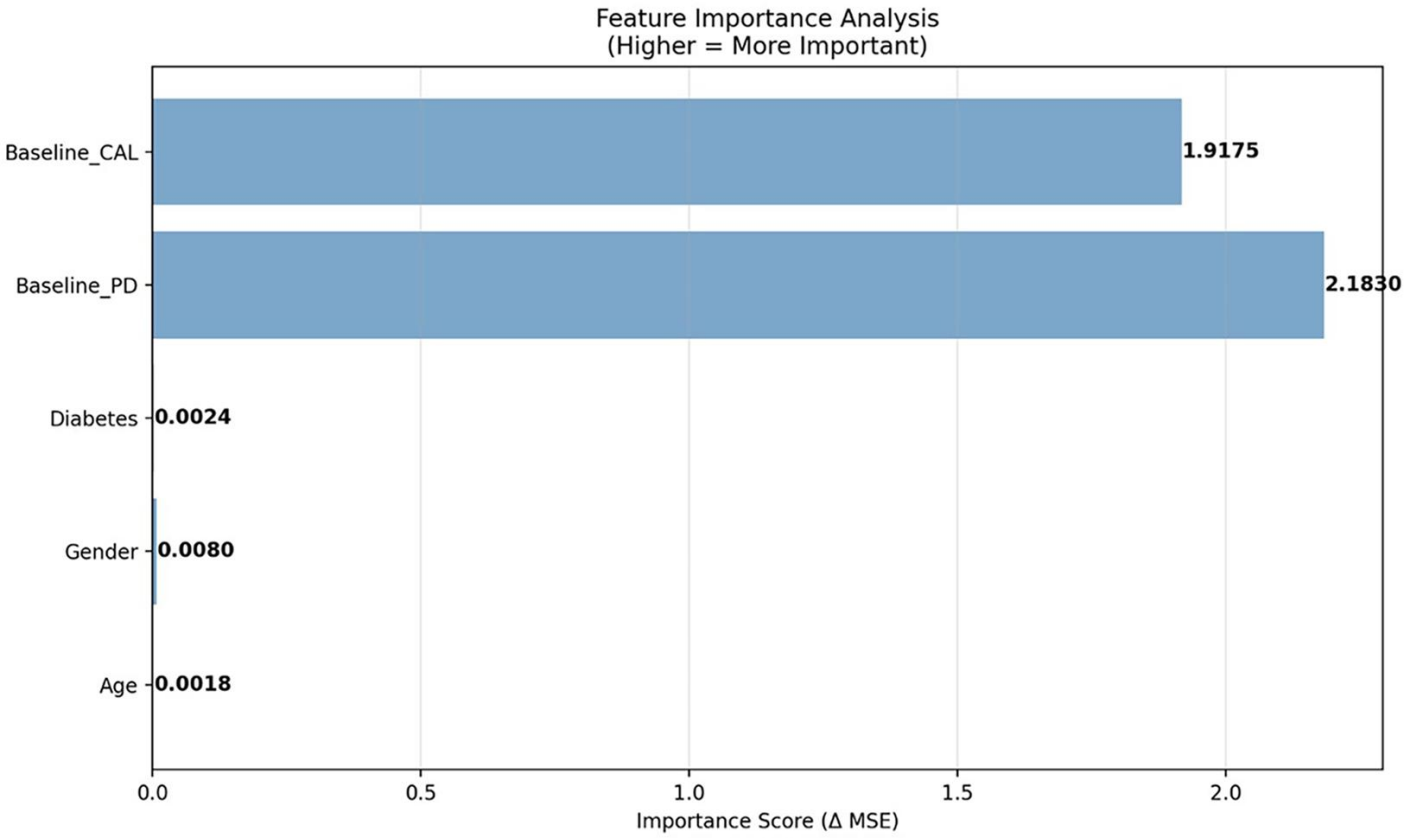
The feature importance analysis of the CaTGO model. Baseline probing depth (PD) and clinical attachment level (CAL) were the primary predictors of outcome. At the same time, age, gender, and diabetes status had a minor influence, indicating that initial disease severity primarily determined the outcome of periodontal healing.

**Figure 6:**
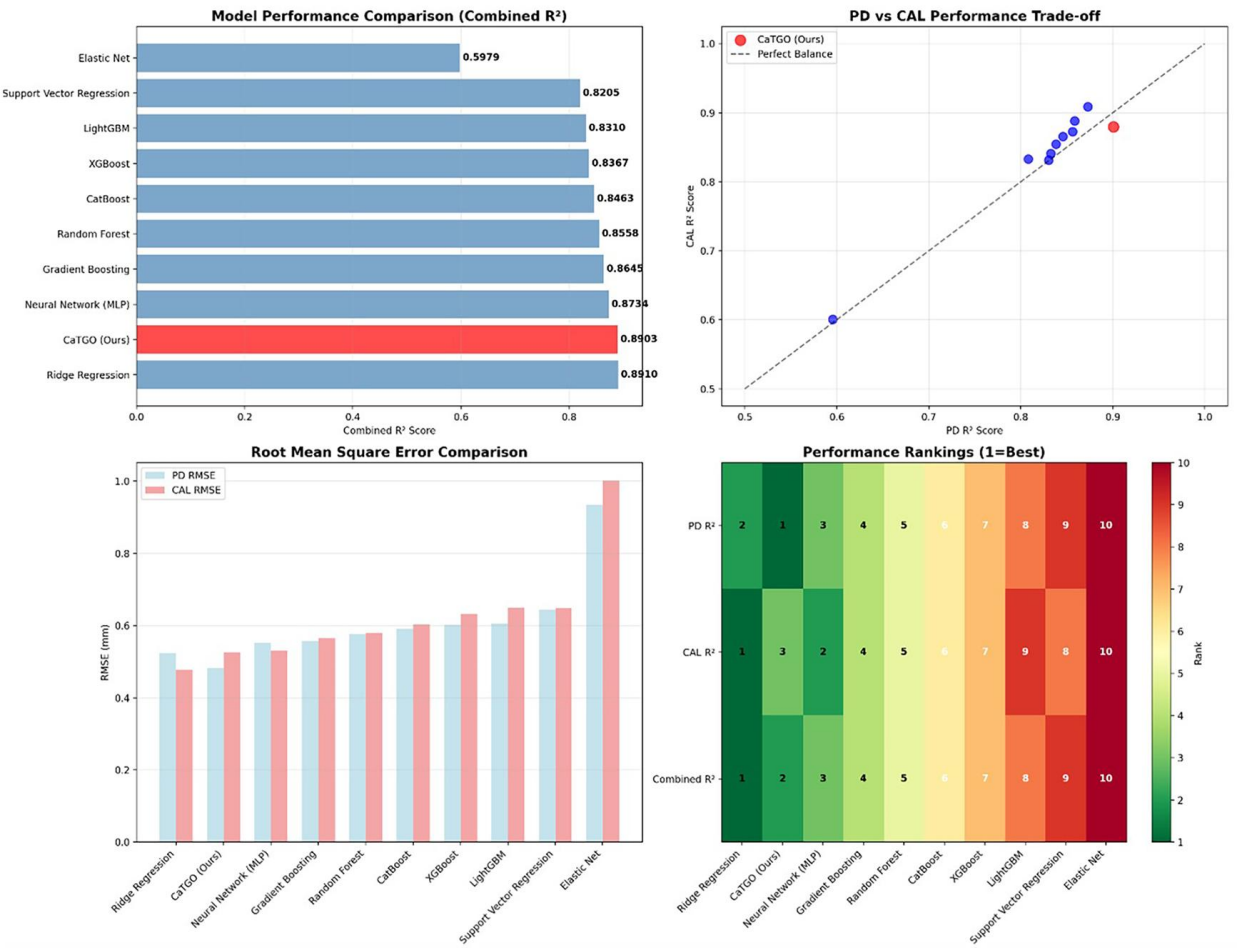
The comparative model performance benchmarking for PD and CAL prediction.

**Figure 7:**
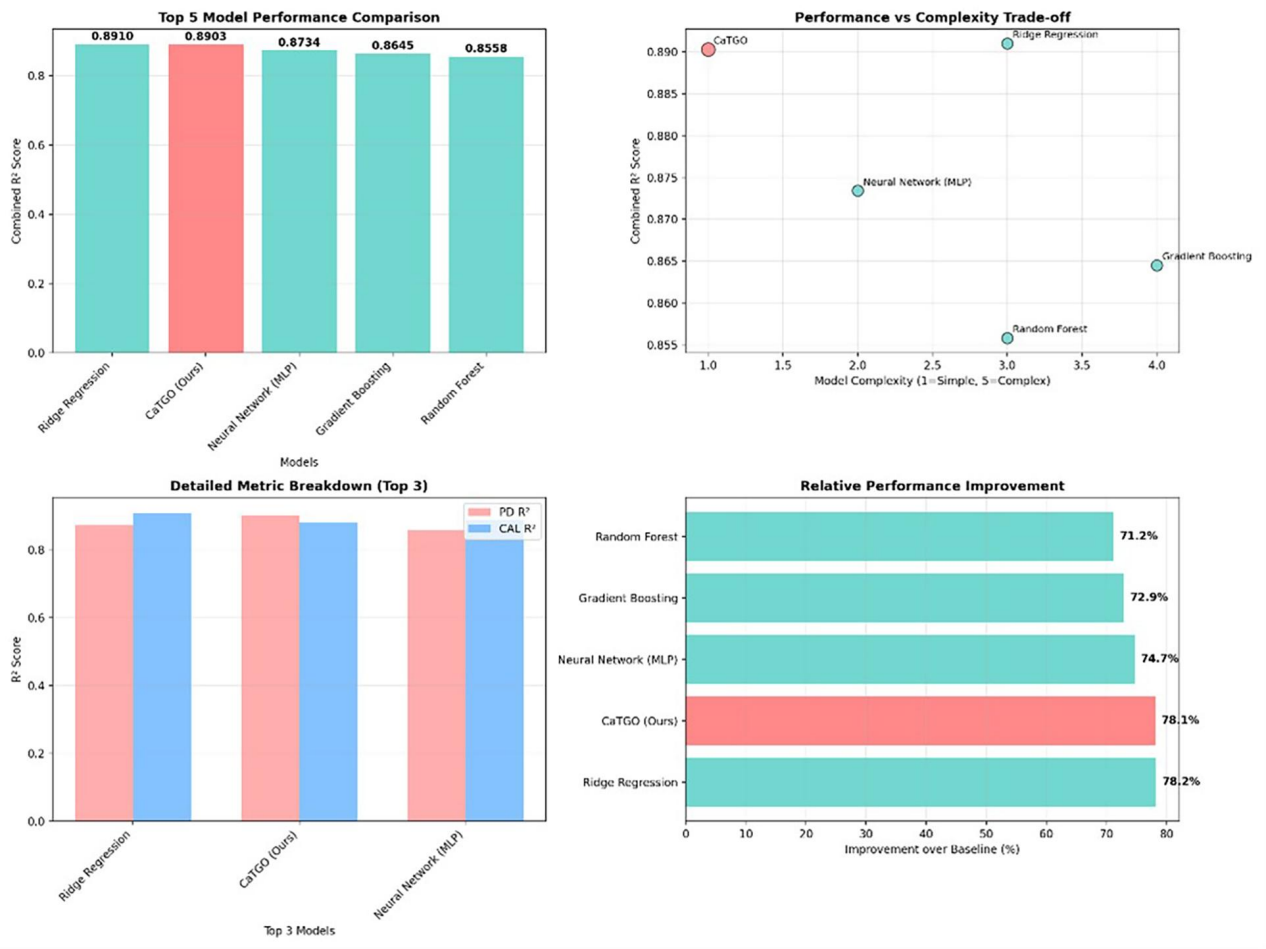
A comparative analysis of top-performing models in predicting periodontal outcomes.

For instance, if the model predicts minimal benefit from LLLT for a particular patient (e.g., a young patient with shallow pockets), the clinician might decide, in consultation with the patient, to forego additional laser sessions, thereby saving time and cost. Conversely, suppose the model identifies a patient (for example, an older diabetic with deep periodontal pockets) as a high responder to LLLT. In such cases, the clinician can confidently recommend adjunctive laser therapy to maximize outcomes, supported by evidence-based justification. Moreover, by quantifying treatment effects, the model facilitates precision periodontics—the tailoring of interventions based on individual risk and expected benefit. This is aligned with the broader movement in medicine towards personalized treatment rather than one-size-fits-all approaches. In a broader healthcare context, models like CaTGO can help allocate resources (like specialized laser equipment and appointments) more efficiently by directing them to cases with the highest payoff, thereby improving overall clinical effectiveness and cost-effectiveness. (Figures 2–7).

From a methodological perspective, our work demonstrates the value of incorporating domain-specific structures (such as tooth graphs) and causal objectives into AI models. The graph representation ensured that predictions reflected the known spatial nature of periodontal disease spread and treatment. At the same time, the causal module worked to address confounding in retrospective data—a common issue that can mislead naive AI models (15–18). By producing models that are not only accurate but also credible and fair (e.g., not systematically biased against certain patient groups) (19, 20).

Dentists should consider diabetic or healing-compromising conditions when recommending LLLT. Due to the strong response, prioritize LLLT for diabetic periodontitis patients unless contraindicated. In mild cases, adjunctive LLLT may be unnecessary for shallow pockets (PD < 4 mm after SRP). This approach improves outcomes by using LLLT where effective and avoiding over-treatment, which prevents patient fatigue, costs, and side effects. Model outputs can also assist communication, e.g., visual outcomes with and without LLLT, to help patients understand the rationale. Diabetic patients generally have worse prognosis; showing them the model predicts better outcomes with LLLT might boost acceptance and adherence.

Our findings are consistent with prior evidence reporting heterogeneous benefits of adjunctive LLLT. The 2017 meta-analysis by Ren et al. demonstrated modest short-term reductions in PD with substantial inter-study variability, whereas the 2021 meta-analysis by Zhao et al. reported more pronounced benefits in metabolically compromised patients, particularly those with diabetes. Recent randomized clinical trials (2013–2025) similarly indicate that adjunctive diode laser therapy may improve probing depth but yields inconsistent CAL gains at the group level. Unlike these studies, which primarily report mean effects, our causal digital twin framework extends this literature by identifying patient- and site-specific heterogeneity, thereby reconciling inconsistent group-level findings through individualized treatment-effect estimation.

Although our results are promising, our study has certain limitations. Being a retrospective analysis, it is susceptible to unmeasured confounding and bias. We addressed this concern through causal modeling, but a prospective, randomized trial is necessary to verify LLLT's effects. Although adjusted for observed variables, treatment effect estimates may still be influenced by unrecorded factors, such as oral hygiene or genetics. Larger, multicenter studies or longer-term outcomes (12 or 24 months) are needed for more robust evidence. The durability of LLLT benefits, especially whether 6-month PD reductions translate into less long-term tooth loss, remains untested. This retrospective single-center study may have residual confounding and measurement variability in PD and CAL assessments despite causal adjustment. Lack of external validation and heterogeneous laser dosing limit generalizability, warranting confirmation in prospective multicenter cohorts.

Future research should assess model performance and LLLT effects over extended periods and broader populations. Our dose-response findings suggest no strong linear relationship within the tested range, but do not rule out the possibility of an optimal dose. We only examined energy density per session; other factors, such as session frequency or total energy, might have non-linear effects. Future research should explore varied protocols to find the optimal balance. Including detailed dose parameters, such as wavelength, power, and duration, could improve results. External validation is necessary; our model demonstrated good internal and cross-validation performance, but it must be tested on independent datasets from different regions or systems to confirm transportability and potential recalibration, thereby strengthening its clinical relevance. Despite being relatively transparent for a deep learning system, it remains a complex entity. Simplifying or integrating it into user-friendly software, such as a chairside tool that inputs patient data and outputs clear predictions with recommendations, is crucial. Future work should include prospective randomized trials with standardized laser parameters to validate CaTGO's causal estimates. External validation on multicenter datasets is needed to confirm generalizability. Incorporating the model into clinical software could enable real-time, personalized support for adjunctive LLLT in routine periodontal care practice (21–23).

## Conclusion

This study shows that a digital twin model accurately predicts periodontal healing and guides personalized therapy after LLLT. The CaTGO model demonstrated high accuracy (R^2^ ∼87%–90%) in predicting changes in pocket depth and attachment level, as well as measuring treatment effects of LLLT. Patients with severe issues benefited most, while those with mild cases gained little. This allows tailored, evidence-based periodontal treatment by matching interventions to patients. The findings support targeted LLLT for high-yield sites to optimize outcomes and resources. Future validation will improve these models and advance personalized care.

## Data Availability

The data analyzed in this study is subject to the following licenses/restrictions: The dataset supporting this study contains clinical records of patients treated in the Department of Periodontics, Saveetha Dental College, and therefore includes potentially identifiable patient information. Due to institutional and ethical restrictions, these data cannot be publicly shared. De-identified data may be made available upon reasonable request to the corresponding author, subject to institutional approval and compliance with data protection regulations. Requests to access these datasets should be directed to pradeepkumar.sdc@saveetha.com.
